# Ribosomal Proteins in Ribosome Assembly

**DOI:** 10.3390/biom15010013

**Published:** 2024-12-26

**Authors:** Brigitte Pertschy, Ingrid Zierler

**Affiliations:** 1 Institute of Molecular Biosciences, University of Graz, Humboldtstrasse 50, 8010 Graz, Austria; 2 BioTechMed-Graz, Mozartgasse 12/II, 8010 Graz, Austria

Ribosomes are the cellular machinery responsible for translating mRNA into proteins, a process fundamental to all domains of life from bacteria to eukaryotes. The synthesis of new ribosomes is a highly orchestrated process involving the assembly of ribosomal proteins with ribosomal RNA (rRNA) into the complex three-dimensional structure of mature ribosomes. This Special Issue focusses on the multifaceted roles of ribosomal proteins in this intricate process, covering topics from their synthesis and transport to their assembly with rRNA, the consequences of their absence, and extra-ribosomal functions.

The current understanding of the role of ribosomal proteins in ribosome assembly is the result of decades of research and technological advances. The review by Gor and Duss (Emerging Quantitative Biochemical, Structural, and Biophysical Methods for Studying Ribosome and Protein–RNA Complex Assembly) provides an overview of the state-of-the-art methods used to study ribosome assembly, focusing on bacteria, and covers some of the methods applied in the research articles within this Special Issue.

But let us move to the beginning (of ribosome synthesis)—in particular, the production of the components for new ribosomes. As well as rRNA, ribosomal protein mRNA also has to be transcribed, and once translated, newly synthesized ribosomal proteins have to be safely transported into the nucleus where the assembly with the rRNA takes place. These early steps of ribosome synthesis are addressed by several articles in this Special Issue.

The study by Di Vona et al. (Loss of the DYRK1A Protein Kinase Results in the Reduction in Ribosomal Protein Gene Expression, Ribosome Mass and Reduced Translation) provides insights into the regulation of ribosomal protein gene transcription in human cells. This study uncovers a novel motif present in a subset of ribosomal protein gene promoters that is bound by the DYRK1A kinase. The presence of DYRK1A enhances the recruitment of the TATA-binding protein, promoting ribosomal protein gene expression. The importance of this novel regulatory pathway is reflected by the observation that the depletion of DYRK1A leads to a global reduction in ribosomal protein gene mRNA, with a concomitant reduction of ribosomal proteins and, consequently, fewer ribosomes.

While translation of ribosomal protein mRNA occurs in the cytoplasm, most newly synthesized ribosomal proteins in eukaryotes must be transported into the nucleus. The research article by Steiner et al. (Dissecting the Nuclear Import of the Ribosomal Protein Rps2 (uS5)) provides insights into how the efficient nuclear import of the ribosomal protein uS5 is achieved in yeast cells. The findings of this study reveal that uS5 possesses at least two nuclear localization sequences (NLSs): an internal NLS recognized by the importin-β Pse1 and an N-terminal NLS. Interestingly, the N-terminal NLS overlaps with the binding site for the uS5-specific chaperone Tsr4, raising the question how the chaperoning and nuclear import of uS5 are coordinated.

Beyond importins and Tsr4, uS5 interacts with multiple additional proteins outside the ribosome. Landry-Voyer et al. (Ribosomal Protein uS5 and Friends: Protein–Protein Interactions Involved in Ribosome Assembly and Beyond) provide an overview of the interaction network of uS5, with a primary focus on the human protein. Human uS5 interacts with the Tsr4 ortholog PDCD2, a dedicated chaperone that protects uS5 from aggregation. Its paralog, PDCD2L, appears to have acquired an independent function, potentially acting as an export adaptor for pre-40S subunits. Furthermore, uS5 is bound by an arginine methyltransferase and a zinc finger protein, although the precise functions of these interactions remain unclear. While some of uS5’s binding partners are involved in ribosome synthesis, others may direct uS5 toward as-yet-uncharacterized extra-ribosomal functions.

An important extra-ribosomal function of many ribosomal proteins is the regulation of p53 signaling in response to impaired ribosome synthesis, a topic studied by Eastham et al. (RPS27a and RPL40, Which Are Produced as Ubiquitin Fusion Proteins, Are Not Essential for p53 Signalling) in human cell lines. RPS27a (eS31) and RPL40 (eL40) are unique among ribosomal proteins as they are synthesized as ubiquitin fusion proteins that require cleavage to release the functional ribosomal protein and ubiquitin. This study investigates whether these two ribosomal proteins, as well as serving as ubiquitin providers, also contribute to p53 signaling. Using several different cell lines, the authors reveal that knockdown of eS31 and eL40 stabilizes p53 in only some of them, suggesting that the involvement of eS31 and eL40 in p53 signaling is cell type specific.

After synthesis of ribosomal proteins, the next critical step is their assembly with the ribosomal RNA as it is transcribed and begins to fold. The article by Rodgers et al. (Ribosomal Protein S12 Hastens Nucleation of Co-Transcriptional Ribosome Assembly) addresses the intricate mechanisms by which one of the early binding ribosomal proteins, S4 (uS4), associates with the ribosomal RNA during transcription. In particular, the authors reveal that one of the later binding proteins, S12 (uS12), accelerates uS4 binding during pre-16S rRNA transcription. This study suggests that uS12 functions as an rRNA chaperone, interacting transiently with a region near the uS4 binding site to facilitate rapid uS4 assembly. It is tempting to speculate that other late binding ribosomal proteins may also play early roles as RNA chaperones.

Beyond RNA folding, the absence of a ribosomal protein can have various effects on ribosome biogenesis, including the inhibition of pre-rRNA processing, or the delayed assembly of other ribosomal proteins or ribosome assembly factors. The review article by Ayers and Woolford (Putting It All Together: The Roles of Ribosomal Proteins in Nucleolar Stages of 60S Ribosomal Assembly in the Yeast *Saccharomyces cerevisiae*) addresses the roles of various ribosomal proteins during the nucleolar stages of 60S ribosomal subunit assembly, focusing on studies of yeast. Taking cryo-EM structural data into consideration, the authors propose several mechanisms for the assembly of ribosomal proteins into nascent ribosomes. They highlight the interplay between neighboring ribosomal proteins and the structural changes of rRNA upon ribosomal protein binding.

The role of ribosomal proteins in the assembly of ribosomal subunits in eukaryotes is also addressed in the review article by Martín-Villanueva et al. (The Beak of Eukaryotic Ribosomes: Life, Work and Miracles), this time focusing on the role of ribosomal proteins that are part of the protrusion of the small subunit termed the “beak structure”. The authors discuss how the composition and structure of the beak have evolved from an all-rRNA structure in bacteria to a more complex arrangement involving multiple ribosomal proteins in eukaryotes and how this structure is assembled.

Having covered the various steps of ribosome assembly in this Special Issue, the last two articles examine how alterations in ribosomal protein composition can influence the structure and function of mature ribosomes. Yu et al. (Structural Insights into the Distortion of the Ribosomal Small Subunit at Different Magnesium Concentrations) investigate the structural dependence of bacterial ribosomes on magnesium ions. A reduction in Mg^2+^ concentration leads to distortions of the decoding center and the dissociation of ribosomal protein S12 (uS12), and it eliminates the interactions of S16 (bS16) with h17, h10, and h15. These findings underscore the critical role of magnesium in maintaining ribosomal integrity, a role that is likely also relevant during ribosome assembly.

Last but not least, the article by Fakih et al. (Differential Participation of Plant Ribosomal Proteins from the Small Ribosomal Subunit in Translation under Stress) addresses how different ribosomal protein isoforms affect translation in plants. This study reveals that specific ribosomal protein isoforms are relevant for the efficient translation of a specific set of mRNAs encoding proteins functioning in pathogen defense. These findings support the hypothesis that plants may utilize functionally specialized ribosomes, incorporating particular ribosomal protein variants to respond effectively to stress conditions.

The research and review articles compiled in this Special Issue offer insights into the multifaceted roles of ribosomal proteins in ribosome biogenesis and beyond. From the regulation of ribosomal protein synthesis and nuclear import to their assembly with rRNA and participation in extra-ribosomal functions, this collection emphasizes the necessity of ribosomal proteins in ribosome biogenesis and function. We hope that these contributions will stimulate further research in the field, advancing our understanding of ribosome biogenesis and its implications for human health and disease.

